# Supplementary material for: An open-source framework for large-scale, flexible evaluation of biomedical text mining systems
Source: J Biomed Discov Collab. 2008 Jan 29;3:1. doi: 10.1186/1747-5333-3-1 (PMC2276192; doi:10.1186/1747-5333-3-1)
Supplement: Additional file 1 — Evaluation platform source code. The additional file contains the software evaluation framework discussed in this paper. The most current release can be downloaded from SourceForge.net [1]. See the accompanying README file for instructions on installation and use. Also included in the distribution is a collection of UIMA wrappers for some commonly used BioNLP tools and annotated corpora. [file 1747-5333-3-1-S1.zip › bionlp_uima_repository-1.0.1/docs/api/src-html/edu/uchsc/ccp/uima/annotation/CCPSpan_Type.html]

```
001    
002    /* First created by JCasGen Tue Aug 28 16:44:10 MDT 2007 */
003    package edu.uchsc.ccp.uima.annotation;
004    
005    import org.apache.uima.cas.Feature;
006    import org.apache.uima.cas.FeatureStructure;
007    import org.apache.uima.cas.Type;
008    import org.apache.uima.cas.impl.CASImpl;
009    import org.apache.uima.cas.impl.FSGenerator;
010    import org.apache.uima.cas.impl.FeatureImpl;
011    import org.apache.uima.cas.impl.TypeImpl;
012    import org.apache.uima.jcas.JCas;
013    import org.apache.uima.jcas.JCasRegistry;
014    import org.apache.uima.jcas.cas.TOP_Type;
015    
016    /** The span object holds span information.This is a supplement to the default UIMA annotation which cannot handle multi-span annotations.
017     * Updated by JCasGen Tue Aug 28 16:44:10 MDT 2007
018     * @generated */
019    public class CCPSpan_Type extends TOP_Type {
020      /** @generated */
021      protected FSGenerator getFSGenerator() {return fsGenerator;}
022      /** @generated */
023      private final FSGenerator fsGenerator = 
024        new FSGenerator() {
025          public FeatureStructure createFS(int addr, CASImpl cas) {
026                             if (CCPSpan_Type.this.useExistingInstance) {
027                               // Return eq fs instance if already created
028                         FeatureStructure fs = CCPSpan_Type.this.jcas.getJfsFromCaddr(addr);
029                         if (null == fs) {
030                           fs = new CCPSpan(addr, CCPSpan_Type.this);
031                               CCPSpan_Type.this.jcas.putJfsFromCaddr(addr, fs);
032                               return fs;
033                         }
034                         return fs;
035            } else return new CCPSpan(addr, CCPSpan_Type.this);
036              }
037        };
038      /** @generated */
039      public final static int typeIndexID = CCPSpan.typeIndexID;
040      /** @generated 
041         @modifiable */
042      public final static boolean featOkTst = JCasRegistry.getFeatOkTst("edu.uchsc.ccp.uima.annotation.CCPSpan");
043     
044      /** @generated */
045      final Feature casFeat_spanStart;
046      /** @generated */
047      final int     casFeatCode_spanStart;
048      /** @generated */ 
049      public int getSpanStart(int addr) {
050            if (featOkTst && casFeat_spanStart == null)
051          jcas.throwFeatMissing("spanStart", "edu.uchsc.ccp.uima.annotation.CCPSpan");
052        return ll_cas.ll_getIntValue(addr, casFeatCode_spanStart);
053      }
054      /** @generated */    
055      public void setSpanStart(int addr, int v) {
056            if (featOkTst && casFeat_spanStart == null)
057          jcas.throwFeatMissing("spanStart", "edu.uchsc.ccp.uima.annotation.CCPSpan");
058        ll_cas.ll_setIntValue(addr, casFeatCode_spanStart, v);}
059        
060      
061     
062      /** @generated */
063      final Feature casFeat_spanEnd;
064      /** @generated */
065      final int     casFeatCode_spanEnd;
066      /** @generated */ 
067      public int getSpanEnd(int addr) {
068            if (featOkTst && casFeat_spanEnd == null)
069          jcas.throwFeatMissing("spanEnd", "edu.uchsc.ccp.uima.annotation.CCPSpan");
070        return ll_cas.ll_getIntValue(addr, casFeatCode_spanEnd);
071      }
072      /** @generated */    
073      public void setSpanEnd(int addr, int v) {
074            if (featOkTst && casFeat_spanEnd == null)
075          jcas.throwFeatMissing("spanEnd", "edu.uchsc.ccp.uima.annotation.CCPSpan");
076        ll_cas.ll_setIntValue(addr, casFeatCode_spanEnd, v);}
077        
078      
079    
080    
081    
082      /** initialize variables to correspond with Cas Type and Features
083            * @generated */
084      public CCPSpan_Type(JCas jcas, Type casType) {
085        super(jcas, casType);
086        casImpl.getFSClassRegistry().addGeneratorForType((TypeImpl)this.casType, getFSGenerator());
087    
088     
089        casFeat_spanStart = jcas.getRequiredFeatureDE(casType, "spanStart", "uima.cas.Integer", featOkTst);
090        casFeatCode_spanStart  = (null == casFeat_spanStart) ? JCas.INVALID_FEATURE_CODE : ((FeatureImpl)casFeat_spanStart).getCode();
091    
092     
093        casFeat_spanEnd = jcas.getRequiredFeatureDE(casType, "spanEnd", "uima.cas.Integer", featOkTst);
094        casFeatCode_spanEnd  = (null == casFeat_spanEnd) ? JCas.INVALID_FEATURE_CODE : ((FeatureImpl)casFeat_spanEnd).getCode();
095    
096      }
097    }
098    
099    
100    
101
```
